# Theoretical studies of magneto-optical Kerr and Faraday effects in two-dimensional second-order topological insulators

**DOI:** 10.1038/s41598-023-39644-y

**Published:** 2023-08-03

**Authors:** Wan-Qing Zhu, Wen-Yu Shan

**Affiliations:** Department of Physics, School of Physics and Materials Science, Guangzhou University, Guangzhou, 510006 China

**Keywords:** Electronic properties and materials, Topological matter, Magneto-optics

## Abstract

Optical approaches are useful for studying the electronic and spin structure of materials. Here, based on the tight-binding model and linear response theory, we investigate the magneto-optical Kerr and Faraday effects in two-dimensional second-order topological insulators (SOTI) with external magnetization. We find that orbital-dependent Zeeman term induces band crossings for SOTI phase, which are absent for trivial phase. In the weak-magnetization regime, these crossings give rise to giant jumps (peaks) of Kerr and Faraday angles (ellipticity) for SOTI phase. In the strong-magnetization regime, we find that two nearly flat bands are formed at the high-symmetry point of Brillouin zone of SOTI phase. These flat bands give rise to two successive giant jumps (peaks) of Kerr and Faraday angles (ellipticity). These phenomena provide new possibilities to characterize and detect the two-dimensional SOTI phase.

In recent years, there has been a surge of interest in the topological properties of quantum materials. Amongst these, the concepts of topological invariants have been generalized to higher orders^1–18^. Different from the conventional correspondence between *d*-dimensional bulk and ($d-1$)-dimensional boundary states in topological insulators, second-order topological insulators (SOTI) have a correspondence between *d*-dimensional bulk and ($d-2$)-dimensional boundary states. For example, in three dimensions ($d=3$), there exist one-dimensional hinge states, which have been observed experimentally in bismuth^8,19^, bismuth halide^20^ and tungsten ditelluride^21^. The roles played by hinge states in physical phenomena have later been revealed, including higher-order interferometer^22^, three-dimensional (3D) quantum Hall and quantum anomalous Hall effect^23,24^, spin transport^25^, etc. By contrast, two-dimensional (2D) SOTI has received relatively less attention due to the difficulties in the material growth and detection of higher-order topology^26–28^.

Optical measurements may provide efficient ways to detect the higher-order topology, as they are bulk sensitive and do not rely on the details of boundary states. When a light is incident into magnetic materials, its angular momentum is transferred to the reflected and transmitted light, respectively, giving rise to the rotations of polarization planes (see Fig. 1). These correspond to the magneto-optical Kerr and Faraday effects, respectively. Such effects have been widely adopted in the detection of time-reversal symmetry breaking in various systems. When applied to 3D topological insulators, quantized and universal Faraday and Kerr rotations have been predicted^29–31^ and experimentally observed^32–34^. Kerr and Faraday effects are not restricted to 3D bulk or film systems, but can be employed to 2D materials. For example, polar Kerr effect may provide fingerprints of spontaneously broken time-reversal symmetry in bilayer graphene^35^. Experimentally, giant Faraday rotations have been observed in monolayer graphene under modest magnetic fields^36,37^. Furthermore, magneto-optical Kerr effects have also been used to experimentally demonstrate the 2D ferromagnetic behaviors of monolayer CrI$_3$^38^ and Cr$_2$Ge$_2$Te$_6$^39^. Since magneto-optical Kerr and Faraday effects can characterize the magnetism and spin behaviors of electrons^40,41^, it motivates us to study the topological properties of 2D SOTI by using these techniques.

In this work, we study the magneto-optical Kerr and Faraday effects in 2D SOTI with out-of-plane magnetization. We consider the generic tight-binding model of 2D SOTI, constructed by the model of 2D topological insulators^2,3,42,43^ with some symmetry-breaking terms. The advantage of the model is that we can switch SOTI phase on and off by tuning parameters. This provides opportunities to compare the results of SOTI with trivial insulators. The light is normally incident into 2D SOTI and magnetic substrate from the vacuum, whose electromagnetic field (also that of reflected or transmitted light) follows the standard Maxwell’s equations^31^. We relate the electromagnetic fields in the vacuum and substrate region by the modified boundary conditions incorporating the conductivities contributed by 2D SOTI. By solving these equations, the Kerr and Faraday angles are then directly obtained from the reflection and transmission coefficients of electric field. On the other hand, the finite-frequency longitudinal and Hall conductivities of 2D SOTI are derived by using the Kubo formula based on linear response theory^44^. Particularly, the Hall conductivity tensor is a consequence of out-of-plane magnetization in 2D SOTI.

For the treatment of magnetization, we consider the Zeeman effect in multi-orbital systems^2,43^, which can be decomposed into orbital-independent and orbital-dependent terms. By symmetry analysis, we find that only orbital-dependent Zeeman term contributes to the Kerr and Faraday effects in such systems. We also find that the magnetization induces band crossings in conduction and valence bands only for the SOTI phase. In the regime of weak magnetization, these crossings lead to giant jumps (peaks) of Kerr and Faraday angles (ellipticity). In the regime of strong magnetization, two nearly flat bands are formed at the high-symmetry *X* point of Brillouin zone of SOTI. These give rise to two successive giant jumps (peaks) of Kerr and Faraday angles (ellipticity) for the SOTI phase. By the quantitative analysis, we find that the model parameters and order of magnitude of rotation angles are all within experimental reach for realistic materials. Therefore these phenomena provide new features to characterize the SOTI phase, which may have practical applications in distinguishing SOTI from trivial insulators.

**Figure 1:**
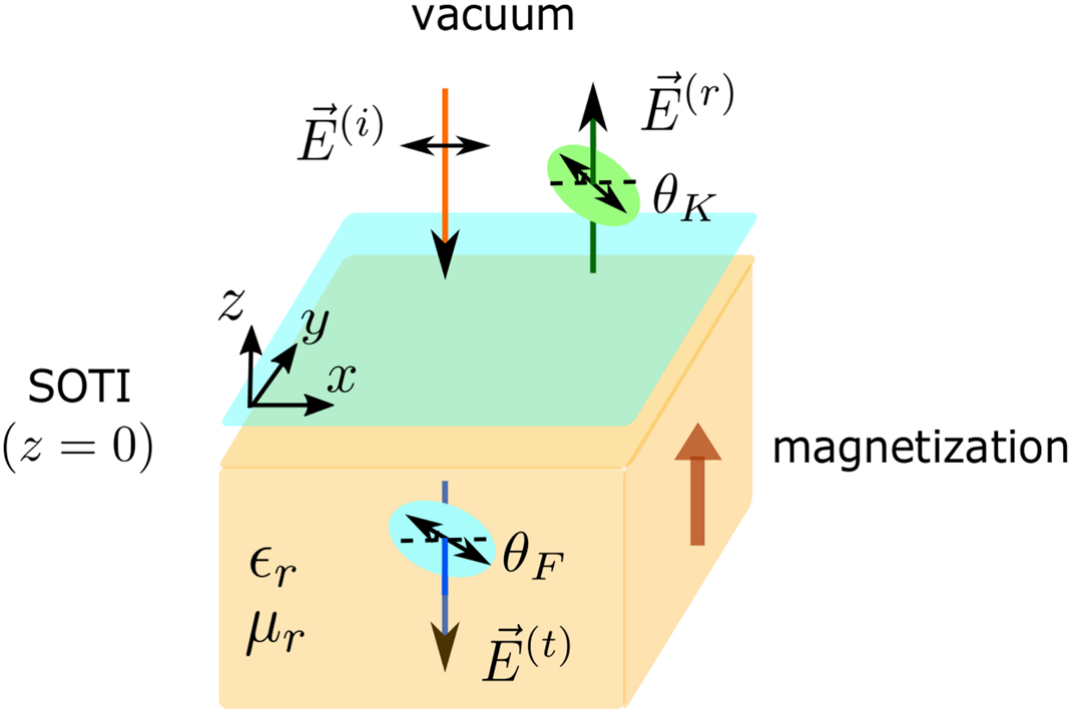
Schematic illustration of magneto-optical Kerr and Faraday effects in 2D second-order topological insulators (SOTI) on a magnetic substrate. $\theta _K$ and $\theta _F$ are Kerr and Faraday angles, respectively. The reflected light is shifted a bit for better visibility.

## Model

We consider a generic tight-binding model of two-dimensional chiral second-order topological insulators $H(\varvec{k})=H_0(\varvec{k})+H_{\varLambda }(\varvec{k})+H_z$^7,9,13^, with

1 $$\begin{aligned} \begin{aligned} H_0(\varvec{k})&= m(\varvec{k})\sigma _z s_0+t \sin (k_xa)\sigma _x s_z+t \sin (k_ya)\sigma _y s_0,\\ H_{\varLambda }(\varvec{k})&= \varLambda (\varvec{k})\sigma _x s_x,\ \ H_z = g\sigma _zs_z. \end{aligned} \end{aligned}$$

Here $m(\varvec{k})=M-2B[2-\sum _{\alpha =x,y}\cos (k_{\alpha }a)]$ and $\varLambda (\varvec{k})=\varLambda [\cos (k_xa)-\cos (k_ya)]$. $k_x$, $k_y$ are the wave vectors and *a* is the lattice spacing (set to be unity). Pauli matrices $\sigma _{\alpha }$ and $s_{\alpha }$ ($\alpha =0,x,y,z$) act on orbital and spin degree of freedoms, respectively. $H_0(\varvec{k})$ is the minimal tight-binding model for topological insulators^3,42^. $H_0(\varvec{k})$ describes topological insulating phase with gapless edge states when $0<M/B<8$; otherwise it describes trivial phase. Here we choose the hopping parameters $t=0.06$ eV adopted from the HgTe quantum well systems^2,43^. Other parameters such as *M* and *B* are discussed in the units of *t* in the following. $H_{\varLambda }(\varvec{k})$ represents a $\hat{\mathcal {T}}$ (time-reversal) symmetry breaking term which gaps the edge states and destroys the topological insulating phase. This leads to the formation of second-order topological insulators.

In this paper, we consider the external magnetization-induced rather than magnetic-field-induced Kerr and Faraday effects. Thus there are no Landau levels and the only consequence of magnetization is the Zeeman energy. For the HgTe quantum wells^2,43^, the Zeeman term reads

2 $$\begin{aligned}H_{zeeman} &= \begin{pmatrix} g_E &{} 0 &{} 0 &{} 0 \\ 0 &{} g_H &{} 0 &{} 0 \\ 0 &{} 0 &{} -g_E &{} 0 \\ 0 &{} 0 &{} 0 &{} -g_H \\ \end{pmatrix}\\&=\frac{g_E+g_H}{2}\sigma _0s_z+\frac{g_E-g_H}{2}\sigma _zs_z. \end{aligned}$$

$g_E$ and $g_H$ originate from different effective *g* factors of electronic orbitals $|E1\rangle$ and $|H1\rangle$. Zeeman term $H_{zeeman}$ can be decomposed into orbital-independent part $\sigma _0s_z$ and orbital-dependent part $\sigma _zs_z$. Here orbital-independent Zeeman term $\sigma _0s_z$ can be neglected since it leads to a zero Hall response. Later we will demonstrate it by symmetry analysis. As a result, we only need to consider the orbital-dependent Zeeman term, which is relabeled by $H_z$ in Eq. (1).

The symmetry properties of the Hamiltonian $H(\varvec{k})$ are summarized in Table 1. In the absence of Zeeman term $H_z$, the Hamiltonian $H(\varvec{k})$ preserves the combined ${\hat{S}}_4={\hat{C}}_4\hat{\mathcal {I}}$ and ${\hat{C}}_4\hat{\mathcal {T}}$ symmetries, whereas breaking $\hat{\mathcal {I}}$, $\hat{\mathcal {T}}$ and ${\hat{C}}_4$ symmetries, respectively. Either $H_z$ or $\sigma _0s_z$ term will break the ${\hat{C}}_4\hat{\mathcal {T}}$ symmetry. Additonally, there is a “hidden” symmetry operation $\hat{\mathcal {P}}=\sigma _xs_y\mathcal {K}$ relating the states with momentum $(k_x,\pm k_y)$, which can be broken by $H_z$ rather than $\sigma _0s_z$. The effect of $\hat{\mathcal {P}}$ on the Hall conductivity $\sigma _{xy}$ will be discussed in the following.

**Table 1:** Symmetry of the Hamiltonian $H(\varvec{k})$ satisfying ${\hat{O}}^{-1}H(\varvec{k}){\hat{O}}=\eta H({\hat{O}}^{-1}\varvec{k})$ with operator ${\hat{O}}$ and $\eta =\pm$. For hidden symmetry operator $\hat{\mathcal {P}}$, $\hat{\mathcal {P}}^{-1}H(\varvec{k})\hat{\mathcal {P}}=\eta H(k_x,-k_y)$.

Symmetry	$\hat{\mathcal {I}}$	$\hat{\mathcal {T}}$	${\hat{C}}_4$	${\hat{S}}_4={\hat{C}}_4\hat{\mathcal {I}}$	${\hat{C}}_4\hat{\mathcal {T}}$	$\hat{\mathcal {P}}$
	$\sigma _zs_0$	$i\sigma _0s_y\mathcal {K}$	$e^{-i\frac{\pi }{4}\sigma _zs_z}$			$\sigma _xs_y\mathcal {K}$
$H_0(\varvec{k})$	$+$	$+$	$+$	$+$	$+$	−
$H_{\varLambda }(\varvec{k})$	−	−	−	$+$	$+$	−
$H_z$	$+$	−	$+$	$+$	−	$+$
$\sigma _0s_z$	$+$	−	$+$	$+$	−	−

The optical conductivity tensor can be given by using the Kubo formula^44^

3 $$\begin{aligned}&\sigma _{\alpha \beta }(\omega )=\\&i\hbar \sum _{\mu \mu^\prime}\int \frac{d^2\varvec{k}}{(2\pi )^2} \frac{f_{\varvec{k}\mu }-f_{\varvec{k} \mu^{\prime}}}{\epsilon _{\varvec{k} \mu }-\epsilon _{\varvec{k}\mu^{\prime}}} \frac{\langle \varvec{k}, \mu |j_\alpha | \varvec{k}, \mu^{\prime} \rangle \langle \varvec{k}, \mu^{\prime} |j_\beta | \varvec{k}, \mu \rangle }{\omega +\epsilon _{\varvec{k} \mu }-\epsilon _{\varvec{k}\mu^{\prime}}+i\hbar / 2\tau _s}, \end{aligned}$$

where $\epsilon _{\varvec{k}\mu }$ and $|\varvec{k},\mu \rangle$ refer to the eigenvalue and eigenstate of Hamiltonian $H(\varvec{k})$ from Eq. (1). $\mu , \mu '=\{1,2,3,4\}$ are band indices. At zero temperature, the Fermi-Dirac distribution $f_{\varvec{k} \mu }=1/[1+\exp ((\epsilon _{\varvec{k}\mu }-\epsilon _F)/k_BT)]=\Theta (\epsilon _F-\epsilon _{\varvec{k}\mu })$, where $\epsilon _F$ is the Fermi energy and $\Theta (...)$ is the Heaviside function. $\omega$ is the photon energy and $\tau _s$ is the relaxation time of bulk states. The contribution of edge states to $\tau _s$ can be safely neglected when the light is shined away from the edge regions. Current operator reads $j_{\alpha }=(e/\hbar )\partial H(\varvec{k})/\partial k_{\alpha }$, with $\alpha ,\beta =\{x, y\}$.

When the orbital-independent Zeeman term $g\sigma _0s_z$ is taken into account, the Hamiltonian $H(\varvec{k})$ preserves the $\hat{\mathcal {P}}$ symmetry (see Table 1). As a result, the eigenstates with momentum $(k_x,\pm k_y)$ satisfy the relations $\epsilon _{\varvec{k}\mu }=-\epsilon _{(k_x,-k_y){\bar{\mu }}}$ and $|\varvec{k},\mu \rangle =e^{i\phi }\hat{\mathcal {P}}|k_x,-k_y,{\bar{\mu }}\rangle$, where $\phi$ is arbitrary phase factor. Moreover, the system has the particle-hole symmetry $\epsilon _{\varvec{k}\mu }=-\epsilon _{\varvec{k}{\bar{\mu }}}$, where $\mu$ and ${\bar{\mu }}$ label a pair of particle-hole-symmetric bands. As an anti-unitary operator, $\hat{\mathcal {P}}$ establishes the following relations between current matrix elements: $\langle \varvec{k},\mu |j_x|\varvec{k},\mu ^{'}\rangle= \langle k_x,-k_y,\bar{\mu ^{'}}|j_x|k_x,-k_y,{\bar{\mu }}\rangle$ and $\langle \varvec{k},\mu |j_y|\varvec{k},\mu ^{'}\rangle= -\langle k_x,-k_y,\bar{\mu ^{'}}|j_y|k_x,-k_y,{\bar{\mu }}\rangle$. To gain some insight, we can decompose the optical conductivity tensor into $\varvec{k}$-resolved components $\sigma _{\alpha \beta }(\omega )=\sum _{\varvec{k}}\sigma _{\alpha \beta }(\varvec{k},\omega )$. As a consequence, we find that $\sigma _{\alpha \alpha }(\varvec{k},\omega )=\sigma _{\alpha \alpha }(k_x,-k_y,\omega )$ for $\alpha =x,y$, and $\sigma _{xy}(\varvec{k},\omega )$ $=-\sigma _{xy}(k_x,-k_y,\omega )$. This indicates that the Hall conductivity $\sigma _{xy}(\omega )=0$ when only the orbital-independent Zeeman term is considered. By contrast, the orbital-dependent Zeeman term $H_z$ breaks the $\hat{\mathcal {P}}$ symmetry, thus gives rise to a nonzero $\sigma _{xy}(\omega )$.

When a light is propagating along $-z$ direction into 2D second-order topological insulators deposited on a magnetic substrate (see Fig. 1), the Kerr and Faraday angles are defined as the relative rotations between left- and right-handed circularly polarized light:^30,31^

4 $$\begin{aligned} \begin{aligned} \theta _K&= \frac{\arg \{E_+^{(r)}\}-\arg \{E_-^{(r)}\}}{2}=\frac{\arg \{r_-\}-\arg \{r_+\}}{2},\\ \theta _F&= \frac{\arg \{E_+^{(t)}\}-\arg \{E_-^{(t)}\}}{2}=\frac{\arg \{t_-\}-\arg \{t_+\}}{2}, \end{aligned} \end{aligned}$$

where the electric field $E_{\pm }^{(l)}=E_{x}^{(l)}\pm iE_{y}^{(l)}$ and $l=r,t$ refer to the reflected and transmitted light, respectively. The reflection (transmission) coefficients read

5 $$\begin{aligned} \begin{aligned} r_{\pm } = \frac{1-\sqrt{\frac{\epsilon _r}{\mu _r}}-Z_0\sigma _{\pm }}{1+\sqrt{\frac{\epsilon _r}{\mu _r}}+Z_0\sigma _{\pm }},\ \ t_{\pm } = \frac{2}{1+\sqrt{\frac{\epsilon _r}{\mu _r}}+Z_0\sigma _{\pm }}, \end{aligned} \end{aligned}$$

where $\sigma _{\pm }=$ $\sigma _{xx}$ ± $i\sigma _{xy}$ and $Z_0=c\mu _0$ $=\sqrt{\mu _0/\epsilon _0}$ $=376.7\Omega$ is the impedance of vacuum. $\epsilon _r$ and $\mu _r$ are the dielectric constant and magnetic permeability, respectively. Then $\theta _K$ and $\theta _F$ can be obtained. See Methods for details of these calculations. Additionally, we can introduce the Kerr and Faraday ellipticity $\gamma _K$, $\gamma _F$:^45^

6 $$\begin{aligned} \begin{aligned} \tan \gamma _K&= \frac{|E_+^{(r)}|-|E_-^{(r)}|}{|E_+^{(r)}|+|E_-^{(r)}|}=\frac{|r_-|-|r_+|}{|r_-|+|r_+|},\\ \tan \gamma _F&= \frac{|E_+^{(t)}|-|E_-^{(t)}|}{|E_+^{(t)}|+|E_-^{(t)}|}=\frac{|t_-|-|t_+|}{|t_-|+|t_+|}. \end{aligned} \end{aligned}$$

By combining $\theta _K$, $\theta _F$ and $\gamma _K$, $\gamma _F$, complex Kerr and Faraday angles can be introduced^40,46^

7 $$\begin{aligned} \begin{aligned} \phi _K&= \theta _K+i\gamma _K,\\ \phi _F&= \theta _F+i\gamma _F. \end{aligned} \end{aligned}$$

**Figure 2:**
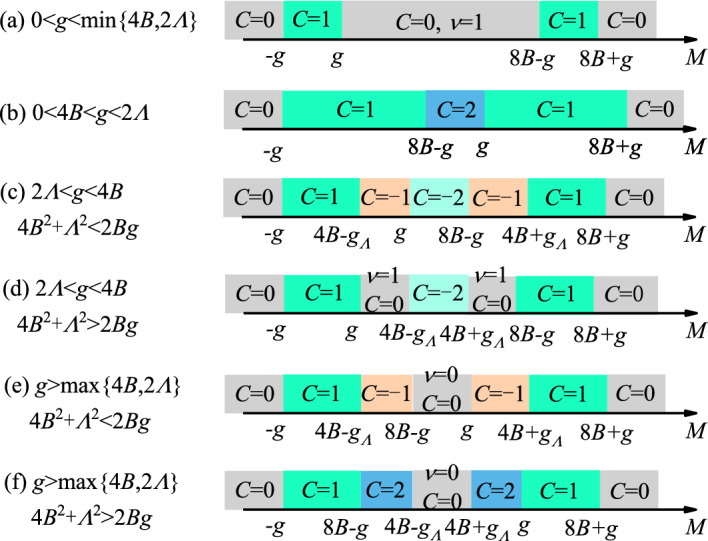
Phase diagram of model Hamiltonian (1) versus *M* for different regimes of parameters. *C* and $\nu$ are the Chern number and second-order topological invariant, respectively. $g_{\varLambda }=\sqrt{g^2-4\varLambda ^2}$.

**Figure 3:**
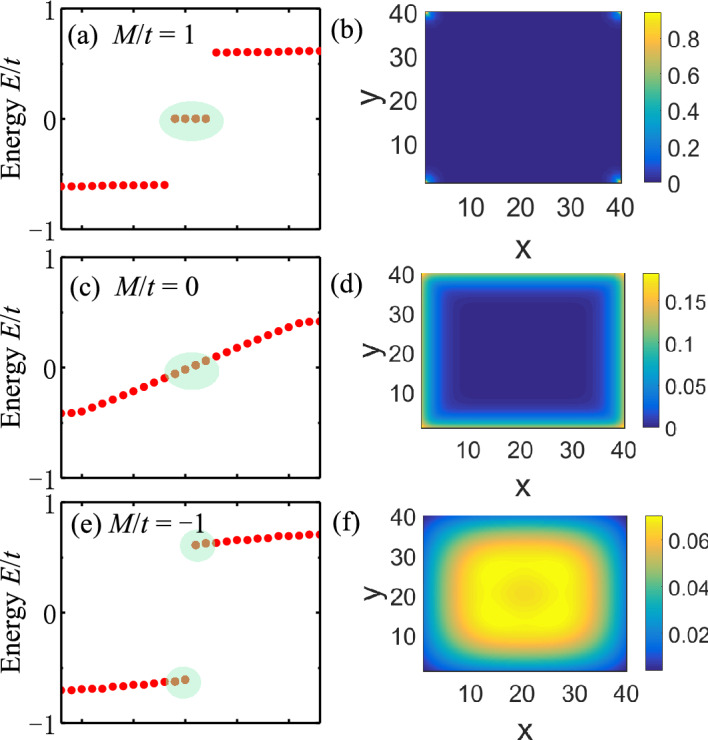
Energy spectrum and wave function distribution of finite-size 2D second-order topological insulators (SOTI) for parameters (**a**), (**b**) $M/t=1$ (SOTI phase), (**c**), (**d**) $M/t=0$ (Chern phase) and (**e**), (**f**) $M/t=-1$ (trivial phase). (**b**), (**d**) and (**f**) denote the summation of distributions $\sqrt{\sum _{i=1}^4|\psi _i|^2}$ of four states highlighted in (**a**), (**c**) and (**e**), respectively. Corner states are present in the four corners of (**b**). Sample size is 40 by 40. Parameters: $t=0.06$ eV^43^, $B/t=0.25$, $\varLambda /t=1.0$, $g/t=0.4$.

## Results

Here we show numerical results of optical conductivities, Kerr and Faraday angles and ellipticity for 2D chiral SOTI in the presence of out-of-plane magnetization. In the absence of magnetization, model Hamiltonian (1) describes a second-order topological phase ($\nu =1$)^9,13,47,48^ when $0<M<8B$ and a topologically trivial phase ($\nu =0$) otherwise. An introduction of magnetization may affect the topological behaviors of the system, thereby inducing a Chern insulating phase^49,50^. The energy dispersions of Hamiltonian (1) are given by

8 $$\begin{aligned} \begin{aligned} \epsilon _{\varvec{k}\mu } = \pm \sqrt{[\sqrt{m^2(\varvec{k})+\varLambda ^2(\varvec{k})}\pm g]^2+t^2\sum _{\alpha }\sin ^2k_{\alpha }}, \end{aligned} \end{aligned}$$

with the band index $\mu =1,2,3,4$. The bulk band gap between two middle bands closes at the high-symmetry momentum $\Gamma =(0,0)$ when $g=|M|$; at $M=(\pi ,\pi )$ when $g=|M-8B|$; at $X=(\pi ,0)$ and $Y=(0,\pi )$ when $g=\sqrt{(M-4B)^2+4\varLambda ^2}$. As a result, diverse topological phases with different Chern number *C* can be realized by tuning the parameters. The phase diagrams of model Hamiltonian (1) are shown in Fig. 2, where both the Chern number *C* and second-order topological invariant $\nu$ are provided. For different regimes of parameters, the phase diagrams can be quite different. To make the discussion explicit, we mainly focus on two regimes of parameters: weak and strong magnetization case, corresponding to case (a) and (e) of Fig. 2.

**Figure 4:**
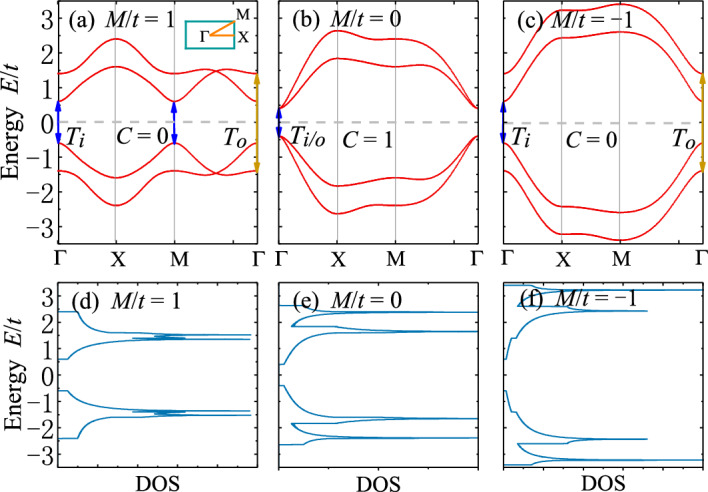
Band dispersions and density of states (DOS) of 2D second-order topological insulators (SOTI) with weak magnetization for parameters (**a**), (**d**) $M/t=1$, (**b**), (**e**) $M/t=0$ and (**c**), (**f**) $M/t=-1$. The dispersions are plotted along the high-symmetry lines of Brillouin zone, as indicated in the inset of (**a**). The optically-induced inner (outer) interband transitions $T_i$ ($T_o$) are depicted by blue (purple) double arrows. In the absence of magnetization, $M/t=1$, 0 and $-1$ correspond to SOTI, semimetal and trivial insulator, respectively. In the presence of magnetization, the Chern number in each case is indicated. Parameters: $t=0.06$ eV^43^, $B/t=0.25$, $\varLambda /t=1.0$, $g/t=0.4$.

**A. Weak magnetization**. First we consider the case with weak magnetization, corresponding to Fig. 2a. In this case, $X=(\pi ,0)$ and $Y=(0,\pi )$ are no longer gap closing points for any given parameters. As a result, the Chern number becomes $C=1$ when $-g< M < g$ or $8B-g< M < 8B+g$, and $C=0$ otherwise. To check the topological properties, we plot the energy spectrum and wave function distribution of finite-size samples in Fig. 3 for parameters: $M/t=1$, 0 and $-1$ with $B/t=0.25$. In the absence of magnetization, these parameters correspond to the SOTI, semimetal and trivial phase, respectively. When magnetization is induced, according to Fig. 2a, these parameters correspond to the SOTI, Chern insulating and trivial phase, respectively. In Fig. 3a and b, we can see the existence of zero-energy corner states. In Fig. 3c and d, we can see the existence of gapless edge states. Such real-space calculations prove our results of phase diagram.

The band dispersions along the high-symmetry lines of Brillouin zone are shown in Fig. 4. Different values of *M* are considered, and Chern numbers are also labeled. Note that the model shows symmetric behaviors between parameters $M>4B$ and $M<4B$, thus we only choose parameters with $M\le 4B$, including $M/t=1$, 0 and $-1$. $T_i$ ($T_o$) labels the optical transitions for two inner (outer) branches of bands. Remarkably, there are new crossings in both conduction and valence bands of SOTI in the $\Gamma -M$ direction (see Fig. 4a), which are absent in the trivial phase. The topological protection of band crossings can be understood by noting that in the $\Gamma -M$ direction (i.e., $k_x=k_y$), $H_{\varLambda }(\varvec{k})=0$ for Hamiltonian (1). Thus the model reduces to that of topological insulators. For topological insulating phase ($0<M<8B$), the bands are inverted at the $\Gamma$ or *M* point, leading to the band crossings between them. For trivial phase, there are no band inversions or crossings.

**Figure 5:**
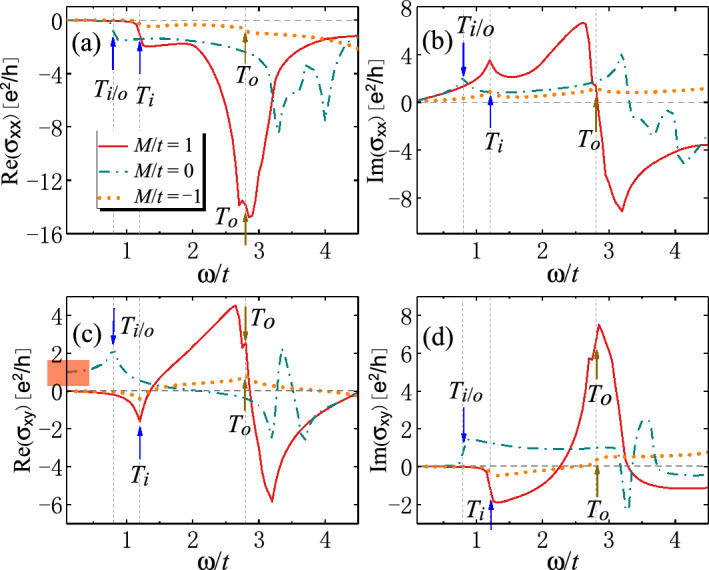
Real and imaginary part of optical conductivities (**a**–**b**) $\sigma _{xx}$ and (**c**–**d**) $\sigma _{xy}$ (in units of $e^2/h$) as functions of photon energy $\omega$ (in units of *t*) for 2D SOTI with weak magnetization. The arrows label the energies of optically-induced inner (outer) interband transitions $T_i$ ($T_o$). The universal value of $\textrm{Re}[\sigma _{xy}]$ in the low-energy limit is highlighted in red in (**c**). Parameters: $t=0.06$ eV^43^, $B/t=0.25$, $\varLambda /t=1.0$, $g/t=0.4$, $\epsilon _r=4$^31,45^, $\mu _r=1$, $\hbar /\tau _s=0.05$, $E_F=0$.

The real and imaginary part of optical conductivities $\sigma _{xx}$ and $\sigma _{xy}$ are plotted in Fig. 5, where for convenience we set the Fermi energy $E_F=0$. A striking difference between SOTI ($M/t=1$) and trivial insulators ($M/t=-1$) lies in their order of magnitude. In SOTI, $\sigma _{xx}$ and $\sigma _{xy}$ are enhanced due to the existence of additional channels of interband transitions. Threshold photon energies for the interband transitions $T_{i}$ and $T_o$ are indicated by arrows in Fig. 5. At these transitions, $\textrm{Re}[\sigma _{xx}]$ and $\textrm{Im}[\sigma _{xy}]$ show sudden jumps while $\textrm{Re}[\sigma _{xy}]$ and $\textrm{Im}[\sigma _{xx}]$ show positive or negative peaks. For example, in Fig. 5a, $\textrm{Re}[\sigma _{xx}]$ show sudden jumps for $M/t=\pm 1$ at $\omega /t=1.2$ due to the activation of inner interband transitions $T_i$. At $\omega /t=2.8$, another jumps occur due to the activation of outer interband transitions $T_o$. For moderate photon energy $\omega$, the magnitude of $\textrm{Re}[\sigma _{xx}]$ for SOTI ($M/t=1$) becomes much larger than trivial insulators ($M/t=-1$). This is attributed to the crossing points along the $\Gamma M$ line of the Brillouin zone of SOTI (see Fig. 4a), which induces new channels of interband transitions at some non-high-symmetry momentum along the $\Gamma M$ line. Moreover, the states at the high-symmetry point $M=(\pi ,\pi )$ have non-negligible contributions due to the band degeneracy between $\Gamma$ and *M*. These together contribute to the large magnitude of $\textrm{Re}[\sigma _{xx}]$ in SOTI. Similar arguments can be given to $\textrm{Im}[\sigma _{xy}]$ (see Fig. 5d). On the other hand, $\textrm{Im}[\sigma _{xx}]$ and $\textrm{Re}[\sigma _{xy}]$ are proportional to the slope of $\textrm{Re}[\sigma _{xx}]$ and $\textrm{Im}[\sigma _{xy}]$, respectively, thereby exhibiting giant jumps near the small peaks at $\omega /t=2.8$ (see Fig. 5b and c). At even higher photon energy, the magnitude of $\sigma _{xx}$ and $\sigma _{xy}$ is greatly reduced due to the closure of optical interband transitions.

**Figure 6:**
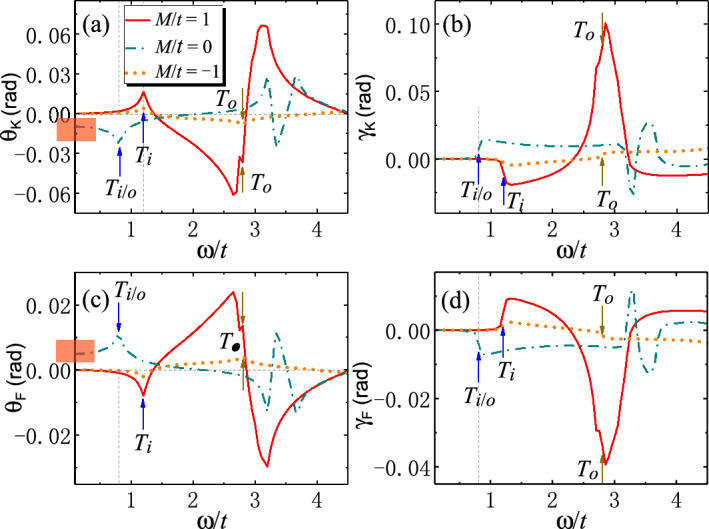
(**a**) Kerr and (**c**) Faraday angles and (**b**) Kerr and (**d**) Faraday ellipticity as functions of photon energy $\omega$ (in units of *t*) for 2D SOTI. The arrows label the energies of optically-induced inner (outer) interband transitions $T_i$ ($T_o$). The universal values of $\theta _K$ and $\theta _F$ in the low-energy limit are highlighted in red in (**a**) and (**c**). Parameters: $t=0.06$ eV^43^, $B/t=0.25$, $\varLambda /t=1.0$, $g/t=0.4$, $\epsilon _r=4$^31,45^, $\mu _r=1$, $\hbar /\tau _s=0.05$, $E_F=0$.

According to Eq. (8), the conditions for the occurrence of crossing points are given by $m(\varvec{k})=\varLambda (\varvec{k})=0$. That is, $k_x=k_y$ ($\Gamma M$ line) and $M-4B[1-\cos (k_{x})]=0$. The critical values of parameters are $M=0$ and $M=8B$, which exactly agree with the parameter range for SOTI. This means that the large magnitude of $\textrm{Re}[\sigma _{xx}]$, $\textrm{Im}[\sigma _{xy}]$ and the giant jump of $\textrm{Im}[\sigma _{xx}]$, $\textrm{Re}[\sigma _{xy}]$ may potentially be used to characterize the SOTI phase. However, such argument is not applicable for the critical value $M=0$, in which case the magnetization drives the system into Chern insulators with Chern number $C=1$ (see Fig. 4b). In this situation, the Chern insulating phase can be distinguished by the integer Hall conductivity in the low-energy limit, that is, $\textrm{Re}[\sigma _{xy}]=e^2/h$ as highlighted in Fig. 5c.

The Kerr and Faraday angles $\theta _K$, $\theta _F$ and ellipticity $\gamma _K$, $\gamma _F$ are plotted in Fig. 6. It is manifest that $\theta _K$ and $\theta _F$ (also $\gamma _K$ and $\gamma _F$) are complementary to each other. Basically, $\theta _F$ ($\theta _K$) shows the same (opposite) behaviors as $\textrm{Re}[\sigma _{xy}]$ in Fig. 5c. This can be understood from Eq. (5), where $Z_0\sigma _{\pm }\ll 1$ can be treated as perturbations. After some algebra, we have $\theta _F\propto -\theta _K\propto \textrm{Re}[\sigma _{xy}]$. This means that Kerr and Faraday angles inherit the properties from Hall conductivity $\textrm{Re}[\sigma _{xy}]$, and hence can also be used to characterize the SOTI. $\gamma _K$ and $\gamma _F$ seem more likely to inherit the properties from $\textrm{Im}[\sigma _{xy}]$, which, together with the Kerr and Faraday angles, can be adopted to distinguish SOTI from trivial insulators.

**Figure 7:**
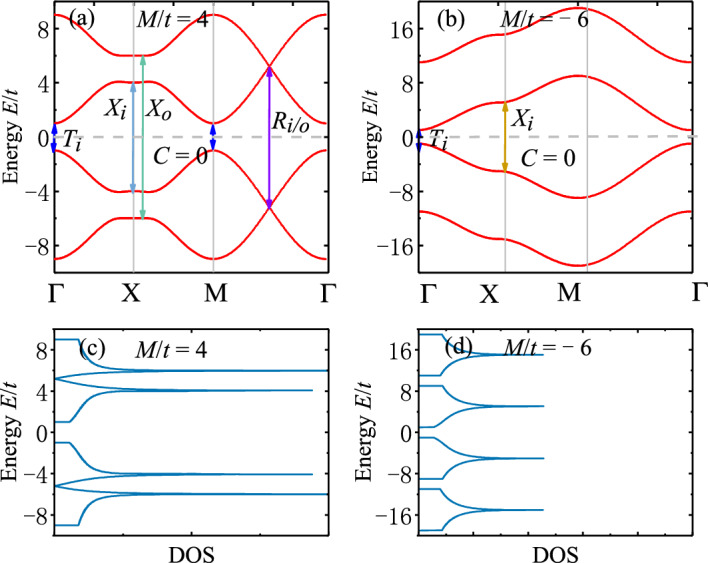
Band dispersions and density of states (DOS) of 2D second-order topological insulators (SOTI) with strong magnetization for parameters (**a**), (**c**) $M/t=4$ and (**b**), (**d**) $M/t=-6$. The optically-induced inner (outer) interband transitions $T_{i/o}$ at $\Gamma$, $X_{i/o}$ at *X* and $R_{i/o}$ at the crossing points are depicted by double arrows. In the absence of magnetization, $M/t=4$ and $-6$ correspond to SOTI and trivial insulator, respectively. In the presence of magnetization, the Chern number in each case is indicated. Parameters: $t=0.06$ eV^43^, $B/t=1$, $\varLambda /t=0.5$, $g/t=5$.

**B. Strong magnetization**. Now we consider the case with strong magnetization, corresponding to Fig. 2e. We consider two representative parameters: $M/t=4$ and $M/t=-6$. In the absence of magnetization, they correspond to the SOTI and trivial phase, respectively. When strong magnetization is induced, the band structure is modified greatly, and $M/t=4$ now reduces to trivial insulating phase. However, we reveal in the following that $M/t=4$ and $M/t=-6$ have distinct optical features as they originate from different topological phases in the absence of magnetization.

The band dispersions along the high-symmetry lines of Brillouin zone are shown in Fig. 7, where two representative parameters are considered: $M/t=4$ (SOTI) and $M/t=-6$ (trivial). We find that similar to the case with weak magnetization, there are crossings in both conduction and valence bands of SOTI, which are absent for trivial phase. The threshold optical transitions for two inner (outer) branches of bands are labeled as $T_{i/o}$ at $\Gamma$ point, $X_{i/o}$ at *X* and $R_{i/o}$ at the crossing points. At the $\Gamma$ or *M* point of Brillouin zone, transitions $T_{i/o}$ are allowed since the initial and final states share the same spin angular momentum. At the *X* point, $H_{\varLambda }(\varvec{k})$ from Eq. (1) mixes states with different spins, despite the fact that inner and outer states are orthogonal to each other. As a result, only transitions $X_{i/o}$ within inner or outer states are allowed.

**Figure 8:**
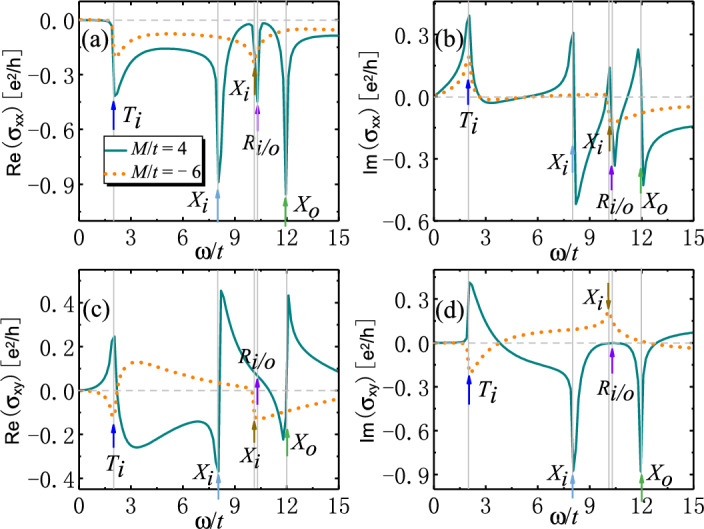
Real and imaginary part of optical conductivities (**a**–**b**) $\sigma _{xx}$ and (**c**–**d**) $\sigma _{xy}$ (in units of $e^2/h$) as functions of photon energy $\omega$ (in units of *t*) for 2D SOTI with strong magnetization. The arrows label the energies of optically-induced inner (outer) interband transitions $T_{i/o}$, $X_{i/o}$ and $R_{i/o}$. Parameters: $t=0.06$ eV^43^, $B/t=1$, $\varLambda /t=0.5$, $g/t=5$, $\epsilon _r=4$^31,45^, $\mu _r=1$, $\hbar /\tau _s=0.05$, $E_F=0$.

The optical conductivities $\sigma _{xx}$ and $\sigma _{xy}$ are plotted in Fig. 8, where the interband transitions contributing to the peaks and jumps are indicated by arrows. We find that SOTI show larger peaks of $\textrm{Re}[\sigma _{xx}]$ and jumps of $\textrm{Im}[\sigma _{xx}]$ from optical transitions $R_{i/o}$ than trivial insulators. Nevertheless, their differences are much smaller than those in the weak-magnetization case. This is due to the loss of degenerate channels of optical transitions driven by strong magnetization. On the other hand, strong magnetization induces nearly flat bands at *X* point for SOTI (see Fig. 7a), which still gives rise to giant peaks and jumps of optical conductivities as a result of the enhanced joint density of states for optical transitions $X_{i/o}$ (see Fig. 7c). This may provide another way to distinguish SOTI from trivial insulators.

**Figure 9:**
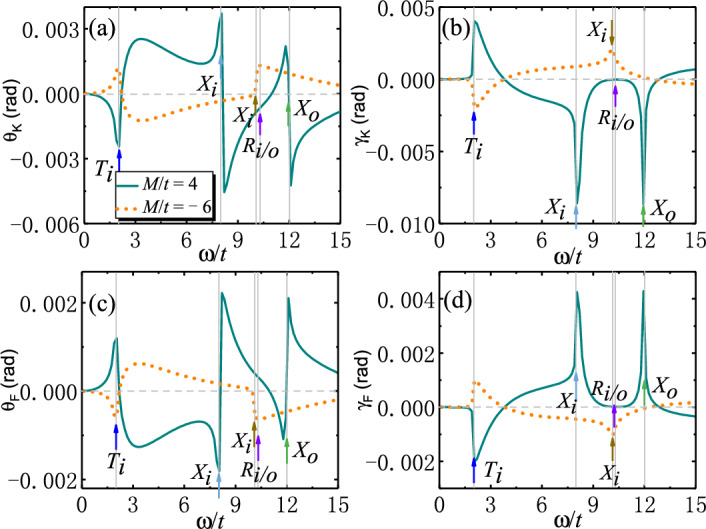
(**a**) Kerr and (**c**) Faraday angles and (**b**) Kerr and (**d**) Faraday ellipticity as functions of photon energy $\omega$ (in units of *t*) for 2D SOTI with strong magnetization. The arrows label the energies of optically-induced inner (outer) interband transitions $T_{i/o}$, $X_{i/o}$ and $R_{i/o}$. Parameters: $t=0.06$ eV^43^, $B/t=1$, $\varLambda /t=0.5$, $g/t=5$, $\epsilon _r=4$^31,45^, $\mu _r=1$, $\hbar /\tau _s=0.05$, $E_F=0$.

The resulting Kerr and Faraday angles and ellipticities are plotted in Fig. 9. There are two successive giant jumps (peaks) in both $\theta _K$ and $\theta _F$ ($\gamma _K$ and $\gamma _F$) originating from optical transitions $X_{i/o}$ for SOTI. By contrast, there is only one small jump or peak from optical transitions $X_{i}$ for trivial insulators. Compared with weak magnetization, strong magnetization tends to suppress the magnitude of Kerr and Faraday angles and ellipticity. The reduction of Kerr and Faraday angles and ellipticity is due to the enhancement of band gaps modified by strong magnetization. This leads to the suppression of optical Hall conductivities, thus the reduction of Kerr and Faraday rotations. This reduction under strong magnetization is different from the general view of magneto-optical effects due to strong magnetic field, where Landau levels are formed. Here the magnetization does not induce Landau levels, but just modifies the band structure. However, even for the reduced Kerr and Faraday angles and ellipticity, they are still within the experimental reach.

## Discussions and conclusions

Numerical results are mainly based on the model parameters of HgTe quantum wells. $t=0.06$ eV and $M/t=1$ are within experimental reach by tuning the quantum well thickness^2,43^. The strong magnetization regime requires that $g/B>4$, suggesting that $g>0.1$ eV. This can be realized in Mn-doped HgTe quantum wells under strong magnetic field^51^, Cr-doped (BiSb)$_2$Te$_3$ thin film^52^ or monolayer MoTe$_2$ on EuO substrate^53^. The photon energy ranges from 0.01 eV to 0.6 eV, corresponding to the terahertz and far infrared frequencies^32–34,54^. In the weak magnetization regime, the rotation angles are tens of mrad, which share the same order of magnitude with experimental results of Bi$_2$Se$_3$ on Al$_2$O$_3$ substrate^32^. In the strong magnetization regime, the rotation angles become a few mrad, in the same order of magnitude with experimental results of strained HgTe and Bi$_2$Se$_3$ on InP substrate^33,34^. Our studies can also be applied to other proposed 2D SOTI, such as graphdiyne^26^, Bi on EuO substrate^27^ and monolayer FeSe^28^.

To realize SOTI from magnetic doped TI, the existence of $H_{\varLambda }(\varvec{k})$ term in Eq. (1) is essential. According to Table  1, $H_{\varLambda }(\varvec{k})$ term breaks $\hat{\mathcal {T}}$ and $\hat{\mathcal {I}}$ symmetries while preserving the ${\hat{C}}_4\hat{\mathcal {T}}$ and ${\hat{C}}_4\hat{\mathcal {I}}$ symmetries. Physically, $H_{\varLambda }(\varvec{k})$ term can be realized in two possible ways. One is to induce orbital currents that break time-reversal symmetry oppositely in the *x* and *y* direction. The other is to induce ($\pi$, $\pi$, 0) noncollinear antiferromagnetic order in the system. Details of this issue are worthy further study.

Note that in our setup (see Fig. 1), semi-infinite magnetic substrate is assumed for simplicity. For realistic samples, the role of substrate thickness should be taken into account. Following the previous work^31^, we know that the results are independent of the substrate properties when the substrate thickness is much smaller than the light wavelength in the low-frequency limit. By increasing the thickness, the magnitude of Kerr and Faraday angles is suppressed. Particularly, when the resonance conditions are satisfied, that is, the substrate thickness contains an integer number of half wavelengths, Kerr and Faraday angles show Fabry-Perot-type oscillations and again become independent of the substrate properties.

Since ellipticity is a dispersive effect and sensitive to the distortions of dielectric tensor, it may not be suitable to detect 2D SOTI. Ellipticity may serve as a supplementary to Kerr and Faraday angles, which together can be used to characterize the 2D SOTI phase. Additionally, the comparison between ellipticity and rotation angles can provide information of distortions or inhomogeneities of the system.

Magneto-optical Kerr and Faraday effects have also been studied in topological insulators^30^ and Floquet topological insulators^55^ . By contrast, we are concentrated on the SOTI with proximity magnetization, rather than introducing the Landau levels^55^. Rashba spin-orbit interaction may further be introduced at the interface as a result of inversion symmetry breaking. This may modify the spin and pseudospin structures of electronic bands, inducing additional channels for optical interband transitions. The presence of impurity scattering affects the relaxation time $\tau _s$ in Eq. (3), which gives rise to a broadening of peaks and jumps for Kerr and Faraday angles and ellipticity^31^. For surface states of topological insulators Bi$_2$Se$_3$ and Bi$_2$Te$_3$^56,57^, hexagonal warping term is present. This term may modify the interband transitions, Fermi velocity and density of states, leading to a quasilinear shape of $\textrm{Re}[\sigma _{xx}]$ with a concave upward bent^58,59^. Our discussion focuses on the zero-temperature limit, and the increasing temperature tends to suppress the magnitude of peaks and jumps of Kerr and Faraday rotations^33^. However, as long as the temperature is not high enough, the main features should still be observable.

To conclude, we have studied the magneto-optical Kerr and Faraday effects in two-dimensional second-order topological insulators. By symmetry analysis, we find that to observe the Kerr and Faraday effects in such systems, Zeeman term must be orbital dependent, rather than orbital independent. The magnetization induces new crossings in conduction and valence bands only in the SOTI phase. In the regime of weak magnetization, these crossings lead to giant peaks of $\textrm{Re}[\sigma _{xx}]$, $\textrm{Im}[\sigma _{xy}]$ and giant jumps of $\textrm{Im}[\sigma _{xx}]$, $\textrm{Re}[\sigma _{xy}]$. As a result, Kerr and Faraday angles (ellipticity) $\theta _K$ and $\theta _F$ ($\gamma _K$ and $\gamma _F$) show giant jumps (peaks) only in the SOTI phase. In the regime of strong magnetization, nearly flat bands are formed at *X* point for SOTI. These give rise to two successive giant peaks of $\textrm{Re}[\sigma _{xx}]$, $\textrm{Im}[\sigma _{xy}]$ and giant jumps of $\textrm{Im}[\sigma _{xx}]$, $\textrm{Re}[\sigma _{xy}]$. In this sense, Kerr and Faraday angles (ellipticity) $\theta _K$ and $\theta _F$ ($\gamma _K$ and $\gamma _F$) show giant jumps (peaks) only in the SOTI phase. These phenomena may potentially be used to distinguish the SOTI from trivial insulators. Note that our proposal may not be applicable to the regime close to the topological phase boundary, such as $M=0$, which may be driven into Chern insulating phase under magnetization.

## Method

We consider a light propagating along $-z$ direction from the vacuum into a 2D material (at $z=0$) deposited on a magnetic substrate (see Fig. 1). In the vacuum ($z>0$), the electric field of incident light reads

9 $$\begin{aligned} \begin{aligned} \varvec{E}^{(i)}(z,t)&= E_x^{(i)}\hat{\varvec{x}}e^{i(-\frac{\omega }{c}z-\omega t)}, \end{aligned} \end{aligned}$$

where $\omega$ and *c* refer to the energy and speed of light in the vaccum, respectively. For the reflected light, the electric field reads

10 $$\begin{aligned} \begin{aligned} \varvec{E}^{(r)}(z,t)&= \left(E_x^{(r)}\hat{\varvec{x}}+E_y^{(r)}\hat{\varvec{y}}\right)e^{i(\frac{\omega }{c}z-\omega t)}. \end{aligned} \end{aligned}$$

In the magnetic substrate ($z<0$), the electric field of transmitted light reads

11 $$\begin{aligned} \begin{aligned} \varvec{E}^{(t)}(z,t)&= \left(E_x^{(t)}\hat{\varvec{x}}+E_y^{(t)}\hat{\varvec{y}}\right)e^{i(-\frac{\omega }{c}n_rz-\omega t)}, \end{aligned} \end{aligned}$$

where the refractive index $n_r=\sqrt{\epsilon _r\mu _r}$. $\epsilon _r$ and $\mu _r$ are the dielectric constant and magnetic permeability, respectively. According to the Faraday’s law $\nabla \times \varvec{E}=-\partial \varvec{B}/\partial t$, the magnetic field of light follows

12 $$\begin{aligned} \begin{aligned} \varvec{B}^{(i)}(z,t)&= -\frac{1}{c}E_x^{(i)}\hat{\varvec{y}}e^{i(-\frac{\omega }{c}z-\omega t)},\\ \varvec{B}^{(r)}(z,t)&= \frac{1}{c}\left(-E_y^{(r)}\hat{\varvec{x}}+E_x^{(r)}\hat{\varvec{y}}\right)e^{i(\frac{\omega }{c}z-\omega t)},\\ \varvec{B}^{(t)}(z,t)&= \frac{n_r}{c}\left(E_y^{(t)}\hat{\varvec{x}}-E_x^{(t)}\hat{\varvec{y}}\right)e^{i(-\frac{\omega }{c}n_rz-\omega t)}. \end{aligned} \end{aligned}$$

Based on the Maxwell’s equations, the boundary conditions at $z=0$ are given by

13 $$\begin{aligned} \begin{aligned} \varvec{E}|_{z=0^+}&= \varvec{E}|_{z=0^-},\\ B_x|_{z=0^-}-B_x|_{z=0^+}&= -\mu _0\mu _r j_y,\\ B_y|_{z=0^-}-B_y|_{z=0^+}&= \mu _0\mu _r j_x, \end{aligned} \end{aligned}$$

where the current density in the 2D material satisfies the relations $j_{\alpha }=\sum _{\beta =x,y}\sigma _{\alpha \beta }E_{\beta }.$ $\epsilon _0$ and $\mu _0$ are vacuum permittivity and permeability, respectively. By substituting the forms of electric and magnetic field into above equations, we can obtain the relations of coefficients from Eqs. (9)–(11).

Now we introduce a scattering matrix between incoming and outgoing electric fields by

14 $$\begin{aligned} \begin{aligned} \left[ \begin{array}{cccc} E_x^{(r)} \\ E_y^{(r)} \\ E_x^{(t)} \\ E_y^{(t)} \\ \end{array}\right] = \left[ \begin{array}{cc} R &{} T' \\ T &{} R' \\ \end{array}\right] \left[ \begin{array}{cccc} E_x^{(i)} \\ 0 \\ 0 \\ 0 \\ \end{array}\right] , \end{aligned} \end{aligned}$$

where

15 $$\begin{aligned} \begin{aligned} R&= \left[ \begin{array}{cc} r_{xx} &{} r_{xy} \\ -r_{xy} &{} r_{yy} \\ \end{array}\right] ,\ \ T = \left[ \begin{array}{cc} t_{xx} &{} t_{xy} \\ -t_{xy} &{} t_{yy} \\ \end{array}\right] \end{aligned} \end{aligned}$$

and similarly for $R'$, $T'$. The detailed form of *R*, *T* can be determined by using the boundary conditions (13). As a result, we find that

16 $$\begin{aligned} \begin{aligned} \left[ \begin{array}{cc} r_{xx} \\ r_{xy} \\ \end{array}\right]&= \frac{1}{D} \left[ \begin{array}{cc} \left(\frac{1}{Z_0})^2-(\frac{1}{Z_0}\sqrt{\frac{\epsilon _r}{\mu _r}}+\sigma _{xx}\right)^2-\sigma _{xy}^2 \\ -\frac{2\sigma _{xy}}{Z_0} \\ \end{array}\right] ,\\ \left[ \begin{array}{cc} t_{xx} \\ t_{xy} \\ \end{array}\right]&= \frac{1}{D} \left[ \begin{array}{cc} \frac{2}{Z_0}\left(\frac{1}{Z_0}+\frac{1}{Z_0}\sqrt{\frac{\epsilon _r}{\mu _r}}+\sigma _{xx}\right) \\ -\frac{2\sigma _{xy}}{Z_0} \end{array}\right] , \end{aligned} \end{aligned}$$

where $D=(\frac{1}{Z_0}+\frac{1}{Z_0}\sqrt{\frac{\epsilon _r}{\mu _r}}+\sigma _{xx})^2+\sigma _{xy}^2$ and $Z_0=c\mu _0=\sqrt{\mu _0/\epsilon _0}=376.7\Omega$ is the impedance of vacuum. In the derivation, we have used the relations $\sigma _{xx}(\omega )=\sigma _{yy}(\omega )$ and $\sigma _{xy}(\omega )=-\sigma _{yx}(\omega )$, which are appropriate for our system. According to the definition of Kerr and Faraday angle from Eq. (4), we need

17 $$\begin{aligned} \begin{aligned} r_{\pm }&= r_{xx}\pm ir_{xy}=\frac{1-\sqrt{\frac{\epsilon _r}{\mu _r}}-Z_0\sigma _{\pm }}{1+\sqrt{\frac{\epsilon _r}{\mu _r}}+Z_0\sigma _{\pm }},\\ t_{\pm }&= t_{xx}\pm it_{xy}=\frac{2}{1+\sqrt{\frac{\epsilon _r}{\mu _r}}+Z_0\sigma _{\pm }}, \end{aligned} \end{aligned}$$

where $\sigma _{\pm }=\sigma _{xx}\pm i\sigma _{xy}$. This reproduces the results in Eq. (5).

## Data Availability

On reasonable request, the corresponding author will provide all relevant data in this paper.
